# Improved assembly and annotation of the sesame genome

**DOI:** 10.1093/dnares/dsac041

**Published:** 2022-11-10

**Authors:** Mingcheng Wang, Jianwei Huang, Song Liu, Xiaofeng Liu, Rui Li, Junjia Luo, Zhixi Fu

**Affiliations:** Institute for Advanced Study, Chengdu University, No. 2025 Chengluo Road, Chengdu 610106, China; Engineering Research Center of Sichuan-Tibet Traditional Medicinal Plant, Chengdu University, Chengdu 610106, China; Berry Genomics Corporation, Beijing 100015, China; Berry Genomics Corporation, Beijing 100015, China; College of Life Sciences, Sichuan Normal University, Chengdu 610101, China; Engineering Research Center of Sichuan-Tibet Traditional Medicinal Plant, Chengdu University, Chengdu 610106, China; School of Food and Biological Engineering, Chengdu University, Chengdu 610106, China; College of Life Sciences, Sichuan Normal University, Chengdu 610101, China; College of Life Sciences, Sichuan Normal University, Chengdu 610101, China

**Keywords:** sesame, oilseed crop, genome assembly, PacBio high-fidelity sequencing, oil metabolism

## Abstract

Sesame (*Sesamum indicum* L.) is an important oilseed crop that produces abundant seed oil and has a pleasant flavor and high nutritional value. To date, several Illumina-based genome assemblies corresponding to different sesame genotypes have been published and widely used in genetic and genomic studies of sesame. However, these assemblies consistently showed low continuity with numerous gaps. Here, we reported a high-quality, reference-level sesame genome assembly by integrating PacBio high-fidelity sequencing and Hi-C technology. Our updated sesame assembly was 309.35 Mb in size with a high chromosome anchoring rate (97.54%) and contig N50 size (13.48 Mb), which were better than previously published genomes. We identified 163.38 Mb repetitive elements and 24,345 high-confidence protein-coding genes in the updated sesame assembly. Comparative genomic analysis showed that sesame shared an ancient whole-genome duplication event with two Lamiales species. A total of 2,782 genes were tandemly duplicated. We also identified several genes that were likely involved in fatty acid and triacylglycerol biosynthesis. Our improved sesame assembly and annotation will facilitate future genetic studies and genomics-assisted breeding of sesame.

## 1. Introduction

Sesame (*Sesamum indicum* L.) is an erect annual herb that belongs to the Pedaliaceae family and has been widely cultivated in tropical and subtropical areas of Asia, Africa, and South America.^1^ It is commonly called “the queen of oilseeds” due to its high seed oil content (~50% of its weight) and quality.^2,3^ Sesame seed oil is a popular edible oil due to its pleasant and mild taste, excellent antioxidant activity, and high nutritional value.^4,5^ Sesame seeds and oil have been widely used in medicinal, cosmetic, and industrial applications,^6^ and have been proposed as a valuable source of biodiesel fuel.^7^ Sesame is one of two oldest oilseed crops known to man (the other is the coconut).^8^ Due to long-term natural and human selection, sesame possesses a high degree of morphological and genetic diversity among cultivated varieties.^9,10^ Elucidating the genetic mechanisms underlying oil-related trait diversity among sesame varieties will benefit the genetic breeding and improvement of this economically important crop.^10,11^

With the rapid development of sequencing technologies, the availability of high-quality reference genomes and genome-wide surveys have provided the opportunity to perform genomics-assisted crop breeding.^12^ Wang et al. first reported an Illumina-based assembly of the sesame genotype, Zhongzhi No. 13, which was 274 Mb in length and 85.3% was anchored to 16 pseudomolecules based on a genetic map.^13^ This reference genome was further improved by a high-density genetic map, where 13 pseudomolecules corresponded with the haploid chromosomal number of sesame (2*n* = 26).^14^ In addition to Zhongzhi No. 13, several Illumina-based genome assemblies corresponding to different sesame genotypes have also been published.^11,15^ These sesame genome sequences have been widely applied in the genetic investigation of various phenotypes by genome-wide association study (GWAS), QTL mapping, gene family analysis, and pan-genome analysis.^10,11,16–19^ However, the publicly available sesame genomes consistently show low continuity with numerous gaps, which may reduce the quality of annotations and lead to missing crucial genomic information.

In this study, we constructed an improved genome assembly of sesame using PacBio high-fidelity (HiFi) reads and Hi-C data. Based on this updated sesame assembly, we generated a high-confidence set of protein-coding genes. We also performed a comparative genomic analysis of sesame and several other sequenced species, as well as investigated the evolutionary history of the sesame genome. The high-quality genome assembly presented here will facilitate future genetic studies and the genomics-assisted breeding of sesame.

## 2. Materials and methods

### 2.1. Sample preparation and sequencing

Fresh leaves were harvested from adult *S. indicum* cv. Baizhima plants grown in Mianyang, Sichuan Province, Southwest China. The variety Baizhima is quite different from other sequenced sesame varieties in several agronomic traits. It has a late flowering habit with increased branches and small white coat seeds. Total genomic DNA was extracted using the cetyl trimethylammonium bromide (CTAB) method^20^ for whole-genome DNA sequencing. First, Illumina ReSeq libraries with an average insert size of 400 bp were prepared and sequenced on an Illumina NovaSeq 6000 platform (Illumina Inc., San Diego, CA, USA). The Illumina sequencing reads were used for the genome survey analysis. Second, SMRTbell libraries were constructed following the PacBio 15-kb protocol and sequenced using the circular consensus sequencing (CCS) mode on a PacBio Sequel II platform (Pacific Biosciences, Menlo Park, CA, USA). The resulting HiFi reads were used for the contig-level genome assembly. Finally, we collected >2 g young leaves and prepared Hi-C libraries following previously described methods,^21^ including chromatin extraction and digestion, DNA ligation, and purification. The Hi-C libraries were sequenced on an Illumina NovaSeq 6000 platform to generate Hi-C reads for the chromosome-level assembly.

In addition to DNA sequencing, total RNA was extracted from fresh leaf, stem, flower, seed, and husk tissues from the same individual sesame plant. RNA-seq libraries were prepared following the TruSeq Stranded mRNA-Seq protocol and sequenced on an Illumina NovaSeq 6000 platform. Low-quality RNA-seq reads were excluded from downstream analysis using Trimmomatic v0.36^22^ with default parameters.

### 2.2. Genome assembly and assessment

Before assembly, the genome size of sesame was estimated based on a 19-mer frequency distribution of Illumina reads generated using Jellyfish v2.2.9.^23^ The homozygous peak depth of *k*-mer distribution was observed and the genome size was calculated using previously described methods with minor modifications.^24^ The HiFi reads were processed using CCS analysis workflow in SMRT Link v8.0 (PacBio) with the following parameters: min-passes = 3 and min-rq = 0.99. The filtered HiFi reads were *de novo* assembled into contigs using hifiasm v0.14^25^ with default parameters. To perform reassignment of allelic contigs caused by highly heterozygous regions, we identified and removed potential duplicate haplotypes from the contig-level assembly using Purge Haplotigs v1.1.1^26^ with the following parameters: read depth low cutoff = 5, low point between the haploid and diploid peaks = 50, and read depth high cutoff = 198. The Hi-C reads were aligned against the draft genome using Juicebox v1.8.8.^27^ Uniquely mapped Hi-C reads were applied to anchor the contigs to pseudochromosomes using 3D-DNA software.^28^ Finally, obvious scaffolding errors were manually adjusted according to the heatmap of chromosome interactions plotted by Juicebox.

To assess the completeness and accuracy of our sesame genome, we mapped the Illumina short reads and RNA-seq reads to the assembly using BWA v0.7.17^29^ and HISAT2 v2.1.0,^30^ respectively. We also calculated the benchmarking universal single-copy orthologs (BUSCO) completeness score of the genome using BUSCO v3.0.2^31^ based on 1,614 conserved genes from the Embryophyta odb10 dataset. Long terminal repeat (LTR) assembly index (LAI) scores were calculated in a 3 Mb sliding window across the entire genome using LTR_retriever v2.8.^32^ Telomere sequences were identified by checking the presence of *Arabidopsis*-type telomeres (TTTAGGG)n in all pseudochromosomes.^33^ Finally, a genome-wide collinearity analysis between our final assembly (hereafter referred to as v3 assembly) and the previously published Zhongzhi 13 assembly (hereafter referred to as v2 assembly) was performed using MUMmer v3.23.^34^

### 2.3. Repeat annotation

The repetitive elements were predicted using RepeatMasker v4.0.7^35^ and RepeatModeler v1.0.11.^36^ A comprehensive repeat library was created by combining the repeats of green plants from the Repbase database v22.11^37^ and the *de novo* repeat library generated by RepeatModeler. Homology searches of the repeats were performed against the genome assembly by RepeatModeler. We identified intact LTR retrotransposons (LTR-RTs) based on their specific structural signals using LTR_Finder v1.06^38^ and LTRharvest v1.5.10.^39^ LTR_retriever was used to integrate the resulting predictions and estimate insertion times of candidate intact LTR-RTs.

### 2.4. Gene prediction

We predicted protein-coding genes within the repeat-masked v3 assembly using previously described methods.^40,41^ We aligned the protein sequences of *Arabidopsis thaliana* TAIR10,^42^*Solanum lycopersicum* ITAG3.2,^43^*Mimulus guttatus* v2.0,^44^*Utricularia gibba* v1.0,^45^*Jacaranda mimosifolia* v1.0,^46^ and *Olea europaea* v1.0^47^ to our v3 assembly using TBLASTN v2.2.31+,^48^ and predicted gene structures using GeneWise v2.4.1^49^ based on the homology alignments. Then, we detected likely protein-coding regions using Program to Assemble Spliced Alignment (PASA) v2.3.3^50^ based on the transcript alignments assembled by *de novo* and genome-guided approaches to the assembly. Next, we performed *de novo* predictions of the genes using Augustus v3.2.3^51^ with parameters trained with selected gene models from the PASA results with an exon number ≥ 3 and CDS length ≥ 1500 bp. Finally, all predicted gene models were integrated into a consensus gene set using EvidenceModeler (EVM) v1.1.1.^52^ The final gene set was searched against the plant transcription factor database (PlantTFDB) v5.0^53^ to identify transcription factor (TF) genes.

In addition to protein-coding genes, we also annotated four types of non-coding RNAs (ncRNAs), including transfer RNA (tRNA), microRNA (miRNA), ribosomal RNA (rRNA), and small nuclear RNA (snRNA) based on structural features and homology searches. The tRNAs were predicted using tRNAscan-SE v2.0^54^ with default parameters. The rRNAs were identified by aligning the publicly available rRNA sequences of *A. thaliana* and *Oryza sativa* to the assembly using BLASTN v2.2.31 with an *E*-value threshold of 1 × 10^-5^. Finally, we annotated the miRNAs and snRNAs by searching against the Rfam database using Infernal v1.1.2.^55^

### 2.5. Functional annotation of protein-coding genes

To assign gene functions, we first aligned the protein sequences against the Swiss-Prot and TrEMBL databases^56^ using DIAMOND v0.9.22.^57^ Conserved protein domains and motifs were identified using InterProScan v5.31.^58^ Gene ontology (GO) IDs were assigned to each gene using Blast2GO v2.5.^59^ The Kyoto Encyclopedia of Genes and Genomes (KEGG) Automatic Annotation Server was used to perform pathway mapping.

### 2.6. Gene family clustering and phylogenetic analysis

The protein sequences of sesame and five other sequenced plant species, *J. mimosifolia*, *M. guttatus*, *O. europaea*, *S. lycopersicum*, and *A. thaliana*, were used for the gene family analysis. These genes were clustered into families using OrthoFinder v2.3.11^60^ with the DIMAOND aligner and Markov cluster (MCL) algorithm. Single-copy orthogroups were extracted from the clustering results and multiple sequence alignment (MSA) was performed for each orthogroup using MAFFT-LINSI v7.313.^61^ A maximum likelihood (ML) phylogenetic tree was constructed based on the concatenated alignments of all single-copy orthogroups using RAxML v8.2.11^62^ under the PROTGAMMAILGX model with 500 bootstrap replicates. Divergence times among the six plant species were estimated using MCMCTREE in PAML v4.9e^63^ with the following parameters: clock = 2, model = 3, and RootAge < 1.25. The divergence time between *A. thaliana* and *S. lycopersicum* (112–125 million years ago (Mya)) was obtained from the TimeTree database (http://www.timetree.org) and used as the fossil calibration point for the MCMCTREE analysis. Gene family expansions and contractions were determined for all internal nodes within the species tree using CAFE v3.1.^64^

### 2.7. Whole-genome and tandem gene duplication analysis

To investigate the whole-genome duplication (WGD) history of sesame and related species, we identified syntenic blocks within and between genomes using MCScanX v1.1^65^ based on the all-against-all pairwise comparisons of protein sequences of *S. indicum*, *J. mimosifolia*, *M. guttatus*, and *O. europaea*. For each ortholog and paralog gene pair, the nonsynonymous substitution rate (*Ka*) and synonymous substitution rate (*Ks*) were calculated using the “add_ka_and_ks_to_collinearity.pl” script in MCScanX. The duplication types were quantified using the “duplicate_gene_classifier” script.

We detected tandem gene duplications by searching homologous gene pairs that: (i) were located within 100 kb of each other; (ii) were located within 10 consecutive genes on a single scaffold; and (iii) had an identity > 50% and a coverage > 70% according to the BLASP results.

### 2.8. Analysis of genes involved in oil metabolism

The oil metabolism genes of sesame were identified by performing homology searches against *Arabidopsis* genes that are involved in fatty acid (FA) and triacylglycerol (TAG) biosynthesis.^66,67^ The expression levels of each gene in the seeds were evaluated using the Fragments per Kilobase per Million (FPKM) values calculated by Cufflinks v2.2.1.^68^ Genes with an FPKM value > 0.5 were considered to be expressed in the seeds.

## 3. Results and discussion

### 3.1. Improvement of the sesame genome assembly

**Figure 1.**
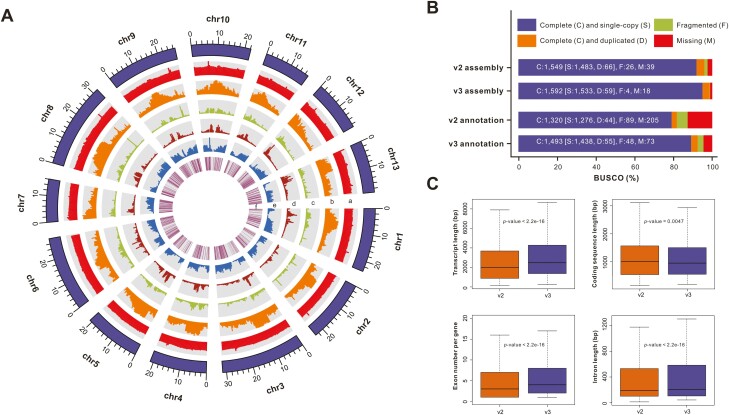
Features of our new sesame assembly and annotation. (A) Landscape of the sesame v3 assembly. Tracks from the outer to inner circles indicate the following: (a) GC content; (b) repeat density; (c) density of *Gypsy* elements; (d) density of *Copia* elements; (e) gene density; and (f) ncRNA locations. (B) BUSCO scores of different sesame assemblies and annotations. (C) Comparisons of protein-coding gene features between the v2 and v3 annotation sets.

A total of 24.33 Gb Illumina reads were generated for the genome survey. The genome size of sesame was estimated to be 317 Mb by the *k*-mer analysis (Supplementary Fig. S1), which was close to the genome size estimation (337 Mb) obtained by flow cytometry in a previous study.^13^ To construct a high-quality, reference-level sesame genome, we generated 34.33 Gb (108.29× genome coverage) HiFi reads with an average read length of 17.1 kb (Supplementary Table S1 and Fig. S2), which were *de novo* assembled into contigs. After discarding potential duplicate haplotypes, we retained 170 contigs with a total length of 309.34 Mb (Supplementary Table S2), covering 97.58% of the estimated genome size. These contigs were anchored to pseudochromosomes based on 32.34 Gb (102.00× genome coverage) Hi-C reads (Supplementary Table S1). In total, 301.73 Mb sequences (97.54% of the entire assembly) were assigned to 13 pseudochromosomes (Fig. 1A; Supplementary Table S3), all of which showed a well-organized diagonal pattern of intra-chromosomal interactions (Supplementary Fig. S3). Compared to the v2 assembly, our v3 assembly showed a 13.42% increase in assembly size (309.35 Mb vs 272.73 Mb), a 288-fold reduction in the number of gaps (35 vs 10,076), and a 254-fold improvement in contiguity (contig N50: 13.48 Mb vs 53.07 kb) (Table 1 and Supplementary Table S2).

**Table 1.** Statistics of the assemblies and annotation sets of the sesame genome

Assembly		
	v2	v3 (This study)
Sequencing platform	Illumina Hiseq 2000	PacBio Sequel II
Assembly size (bp)	272,734,981	309,354,609
GC (%)	34.98	35.44
Number of scaffolds	4,449	135
Scaffold N50 size (bp)	20,257,639	23,373,591
Longest scaffold length (bp)	26,180,356	31,985,284
Number of contigs	14,525	170
Contig N50 size (bp)	53,067	13,482,096
Longest contig length (bp)	471,223	31,438,572
Number of gaps	10,076	35
BUSCO score (%)	95.97	98.64
Annotation		
	v2	v3 (This study)
Repeat content (%)	40.26	52.81
Transposable elements (%)	21.59	27.42
Unclassified repeats (%)	18.66	26.30
Protein-coding genes		
Total number	25,172	24,345
Average gene length (bp)	2,891	3,422
Average exon per gene	4.86	5.98
Average CDS length (bp)	1,207	1,206
Average intron length (bp)	436	445
BUSCO score (%)	81.78	92.50

The high fidelity of our sesame v3 assembly was supported by the high mapping rate (99.57%) and 10-fold minimum genome coverage (99.13%) of the Illumina reads (Supplementary Table S4). The high completeness of our v3 assembly was also evidenced by the high mapping rate (94.56%) of the RNA-seq reads (Supplementary Table S5). Furthermore, we observed a BUSCO completeness score of 98.64% (Fig. 1B; Table 1) and an average LAI score of 19.22 for the entire assembly, which were better than the v2 assembly (BUSCO completeness score: 95.97%; LAI score: 13.92) (Fig. 1B; Supplementary Fig. S4). We found that 11 pseudochromosomes contained telomere sequences at both ends, and 2 pseudochromosomes contained telomere sequences at a single end, suggesting the good integrity of our v3 assembly (Supplementary Table S6). The MUMmer alignment analysis showed that the global collinearity was mostly conserved between the v2 and v3 assemblies (Supplementary Fig. S5) except for a large inversion corresponding to 8.48 Mb of genome sequence in “chr12” of the v3 assembly, indicating the high accuracy of the Hi-C scaffolding analysis. Several central regions of pseudochromosomes in the v3 assembly showed no similarity to any region of the v2 assembly and exhibited high levels of repetitive elements (Supplementary Table S7), indicating the advantage of PacBio sequencing in the assembly of highly repetitive regions. Collectively, these metrics indicated the high quality and completeness of our updated sesame assembly.

### 3.2. Updated annotation of the sesame genome

Using a combination of *de novo* and homology-based approaches, we identified 163.38 Mb repetitive elements representing 52.81% of the v3 assembly, which was much higher than the repeat content re-identified in the v2 assembly (109.80 Mb; 40.26% of the entire genome) (Table 1; Supplementary Table S8). Transposable elements (TEs) were the most abundant repeat class, spanning 84.84 Mb (27.42%) of the updated assembly with LTR-RTs being the dominant type (44.36 Mb), followed by long interspersed nuclear elements (LINEs; 24.06 Mb), DNA transposons (16.28 Mb), and short interspersed nuclear elements (SINEs; 137.88 kb). *Copia* and *Gypsy* were the two most abundant LTR-RTs, representing 7.12% and 6.61% of the entire assembly, respectively. The divergence rate distribution analysis showed that most (70.83%) of the TEs had a divergence rate of < 20% (Supplementary Fig. S6). To gain further insight into the TE amplification dynamics in sesame, we identified 778 intact LTR-RTs, including 417 *Copia* and 355 *Gypsy* elements. Most of the intact LTR-RTs (58.74%) were younger than 2 million years, with median insertion times of 1.03 and 1.52 Mya for the *Copia* and *Gypsy* elements, respectively (Supplementary Fig. S7). In addition to TEs, we identified 81.37 Mb (26.30% of the genome) unclassified repetitive sequences, 65.45% of which had a divergence rate of < 20% (Supplementary Fig. S8). Overall, the repetitive elements accumulated gradually with high recent activity within the sesame genome.

Combing *de novo* predictions, homology searches, and transcript evidence from the RNA-seq data, we identified 24,345 protein-coding genes, 23,947 (98.37%) of which were located on pseudochromosomes with an overall gene density of 79.4 gene per Mb (Supplementary Table S3). Overall, 21,916 (90.02%) protein-coding genes were functionally annotated by the Swiss-Prot and TrEMBL databases, 23,051 (94.68%) genes contained conserved domains recognized by the InterProScan analysis, 18,722 (76.90%) genes were assigned to GO terms, and 8,270 (33.97%) genes were mapped to KEGG pathways (Supplementary Table S9). Within the final gene set, we identified 1,223 TF genes belonging to 57 subfamilies (Supplementary Table S10). In addition to protein-coding genes, we identified 1,488 tRNAs, 129 miRNAs, 2,002 rRNAs and 350 snRNAs in the sesame genome (Supplementary Table S11).

Compared to the updated gene annotation set of the v1 assembly (hereafter referred to as v2 annotation), which was retrieved from the Ensembl Plants portal (http://www.plants.ensembl.org), our updated annotation set (hereafter referred to as v3 annotation) showed higher BUSCO completeness score (92.50% vs 81.78%) (Fig. 1B; Table 1), a longer average transcript length (3,422 bp vs 2,891 bp), similar average coding sequence (CDS) size (1,206 bp vs 1,207 bp), more exons per gene (5.98 vs 4.86), and a longer average intron length (445 bp vs 436 bp) (Table 1 and Fig. 1C). Among the 25,172 protein-coding genes in the v2 annotation set, 1,907 had no significant BLASTP hits to any annotated gene in our v3 annotation set, of which 1,534 (80.44%) did not have any functional annotation. These genes were significantly shorter than the other genes in the v2 annotation set (Wilcoxon rank sum test, *p*-value < 2.2e-16) (Supplementary Fig. S9). Therefore, we speculated that these genes may have been falsely annotated. Conversely, we identified 1,822 genes in our v3 annotation set that were missing from the v2 annotation set, of which 1,440 (79.03%) were supported by the functional annotation or gene expression analysis (Supplementary Fig. S10). Furthermore, we identified 13,973 1:1 orthologous gene pairs between the sesame v2 and v3 annotation sets by the MCScanX analysis, of which 7,138 were longer in our v3 annotation set than in the v2 annotation set, indicating that these genes may have been partially annotated in the previous assembly. Collectively, these findings indicated that our high-quality sesame assembly had more accurate structural annotations of the protein-coding genes.

### 3.3. Genome evolution of sesame

**Figure 2.**
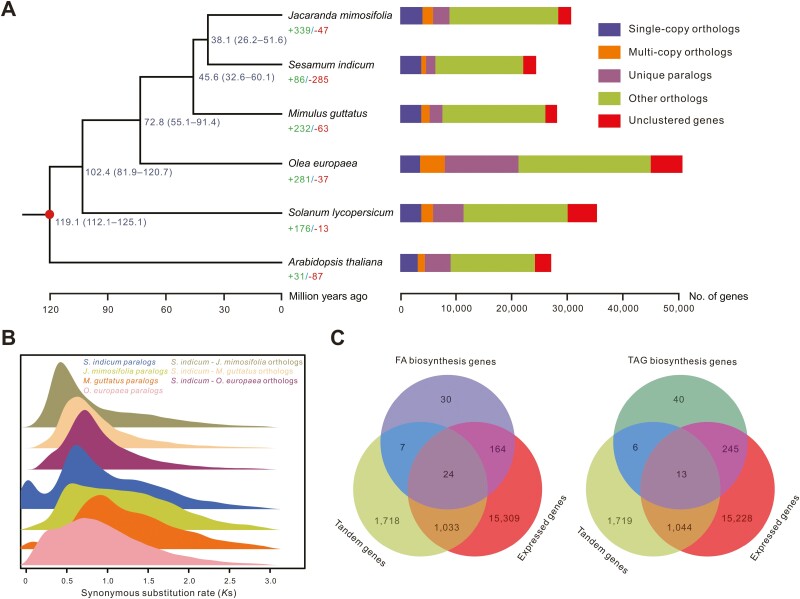
Genome evolutionary analysis of sesame. (A) Fully supported species phylogeny based on 2,093 single-copy orthogroups from sesame and five other plant species with gene family clusters. The circle represents the fossil calibration point. Gene family expansions and contractions are marked with “+” and “−”, respectively. Divergence times with 95% confidence intervals are located on each internal node. (B) *K*s distributions of the whole paranome identified from the whole genome of *S. indicum*, *J. mimosifolia*, *M. guttatus*, and *O. europaea*. (C) Venn diagram of FA biosynthesis genes, TAG biosynthesis genes, tandemly duplicated genes, and genes expressed in the seeds.

To investigate the evolutionary dynamics of the gene families in sesame, we clustered 22,044 (90.55%) sesame genes into 14,173 families by the OrthoFinder analysis (Fig. 2A). Among which, 9,798 (69.13%) families were shared by all six plant species and 334 families (containing 1,597 genes) were unique to sesame. The GO enrichment analysis of these sesame specific genes showed that they were highly enriched in “proton export across plasma membrane” (GO:0120029), “carbohydrate metabolic process” (GO:0005975), “regulation of intracellular pH” (GO:0051453), and “organic substance metabolic process” (GO:0071704) (Supplementary Fig. S11). Then, we constructed a fully supported species tree based on 2,093 single-copy orthogroups from the six species (Fig. 2A). The ML phylogenetic tree showed that *S. indicum* clustered together with *J. mimosifolia*, which was consistent with the species phylogeny of a previous study.^46^ The divergence time between these two species was estimated to be ~38 Mya (26–52 Mya). We also identified 86 gene families comprised of 606 genes that were expanded in sesame (*P* < 0.05), which were functionally enriched in “translation” (GO:0006412), “nucleic acid phosphodiester bond hydrolysis” (GO:0090305), “mRNA processing” (GO:0006397), “ion transport” (GO:0006811), and “oligopeptide transport” (GO:0006857) (Supplementary Fig. S12). Based on the MCScanX results, 447 (73.76%) genes were located within the expanded families and classified as “tandem” (121, 19.97%) and “WGD/segmental” (326, 53.80%) (Supplementary Fig. S13).

To elucidate the polyploidy history of sesame and related species, we examined the *K*s distributions of the paralogs and orthologs identified from *S. indicum*, *J. mimosifolia*, *M. guttatus*, and *O. europaea* (Fig. 2B). We observed a major peak around 0.43 in the *K*s distribution of orthologs between *S. indicum* and *J. mimosifolia*, which was younger than the two peaks identified in the paralog analysis of *S. indicum* (0.62) and *J. mimosifolia* (0.57). Additionally, the *K*s peak of the orthologs between *S. indicum* and *M. guttatus* (0.63) was younger than the *K*s peak of paralogs within *M. guttatus* (0.92), and two *K*s peaks of paralogs within *O. europaea* (0.24 and 0.71) were younger than the *K*s peak of orthologs between *S. indicum* and *O. europaea* (0.72). Therefore, we speculated that a shared WGD event had occurred in the common ancestor of *S. indicum*, *J. mimosifolia*, and *M. guttatus*, and that two independent WGD events had occurred in *O. europaea*, which was consistent with a previous report.^47^ Based on the peaks of *K*s distribution and divergence time between *S. indicum* and *J. mimosifolia*, the shared WGD event likely occurred between 50.8 and 55.3 Mya. In addition to the WGD, we identified a total of 886 tandem arrays containing 2,782 genes within the sesame genome (Supplementary Table S12). These tandemly duplicated genes were functionally related to “terpenoid biosynthetic process” (GO:0016114), “nucleic acid phosphodiester bond hydrolysis” (GO:0090305), “coumarin biosynthetic process” (GO:0009805), “phosphate ion transport” (GO:0006817), and “carbohydrate metabolic process” (GO:0005975) (Supplementary Fig. S14).

Within the updated sesame v3 genome, we identified 225 genes that were possibly related to FA biosynthesis, of which 188 (83.56%) were expressed in the seeds and 31 (13.78%) were tandemly duplicated (Fig. 2C). We also identified 304 genes that were possibly involved in TAG biosynthesis, of which 258 (84.87%) were expressed in the seeds and 19 (6.25%) were tandemly duplicated (Fig. 2C). These candidate genes provide essential information for future genetic investigations on oil-related traits in sesame, which may be useful for the genomics-assisted breeding of this economically important crop.

## Conclusions

In this study, we reported an updated sesame assembly based on highly accurate, long-read HiFi sequencing and Hi-C sequencing data. Compared to the previously published Zhongzhi 13 assembly, our sesame v3 assembly had significantly higher contiguity and completeness. Based on this high-quality assembly, we predicted 24,345 high-confidence protein-coding genes with more accurate structural annotation. Evolutionary analysis showed that *S. indicum* and *J. mimosifolia* clustered together and these two species shared an ancient WGD event with *M. guttatus*. Additionally, we found that > 10% of the protein-coding genes within the sesame genome were tandemly duplicated. We also identified several genes that were possibly involved in oil metabolism. Our high-quality, reference-level sesame assembly and improved genome annotations lay the foundation for elucidating the genetic basis of oil metabolism, abiotic stress resistance, and other key agronomic traits.

## Supplementary Material

dsac041_suppl_Supplementary_Material

## Data Availability

The sesame v3 assembly and all sequence data are available in the NCBI database under the BioProject PRJNA875260. Genome assembly has been deposited at DDBJ/ENA/GenBank under the accession JAOXLQ000000000. RNA-seq data are available under the Sequence Read Archive (SRA) accession numbers SRX17378544-SRX17378548. Illumina short-read data, HiFi long-read data, and Hi-C data are available under the SRA accession numbers SRX17389771, SRX17602893, and SRX17602393. The updated genome assembly and annotations are also available on FigShare (https://doi.org/10.6084/m9.figshare.21151948).
