# Supplementary material for: Improved assembly and annotation of the sesame genome
Source: DNA Res. 2022 Nov 10;29(6):dsac041. doi: 10.1093/dnares/dsac041 (PMC9724774; doi:10.1093/dnares/dsac041)
Supplement: dsac041_suppl_Supplementary_Material [file dsac041_suppl_supplementary_material.docx]

**
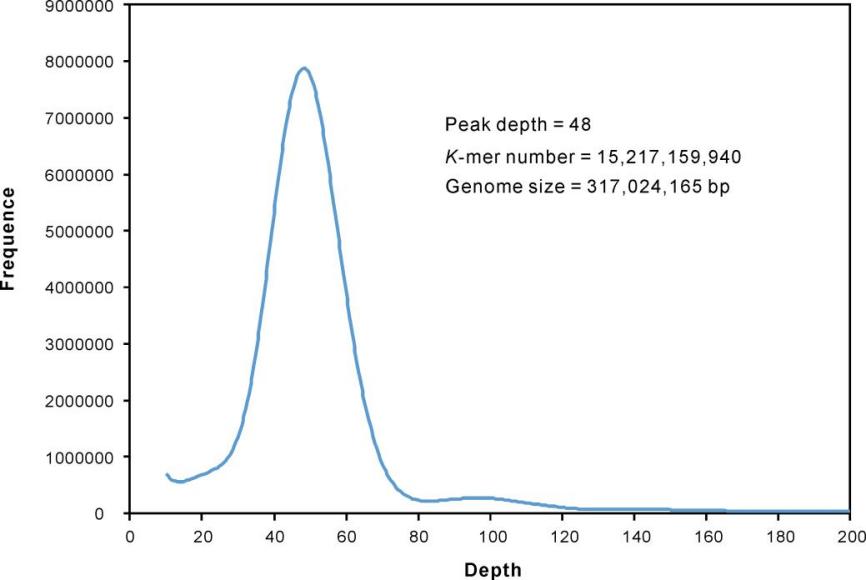
**

**Supplementary Figure S1. A 19-mer frequency distribution of sesame based on Illumina short reads.**

**
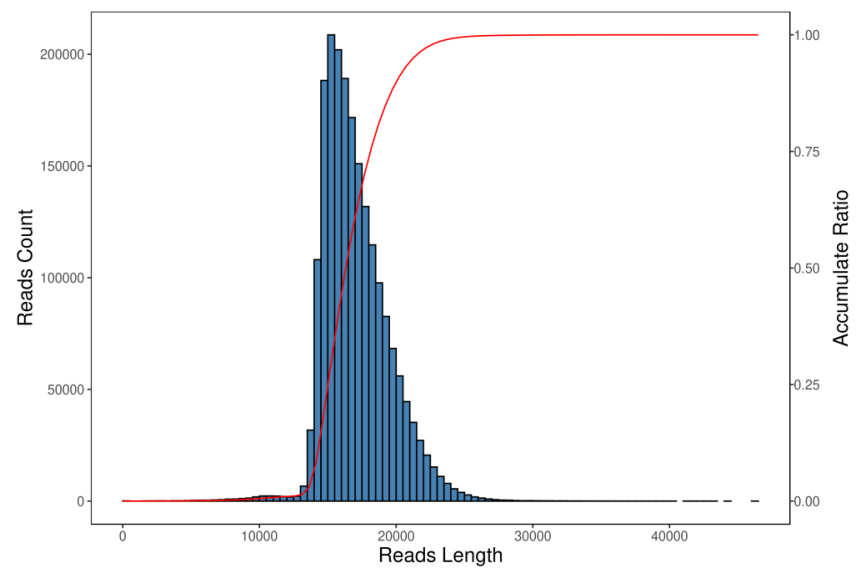
**

**Supplementary Figure S2. Plot of sub-reads length distribution of PacBio’s HiFi sequencing data.**


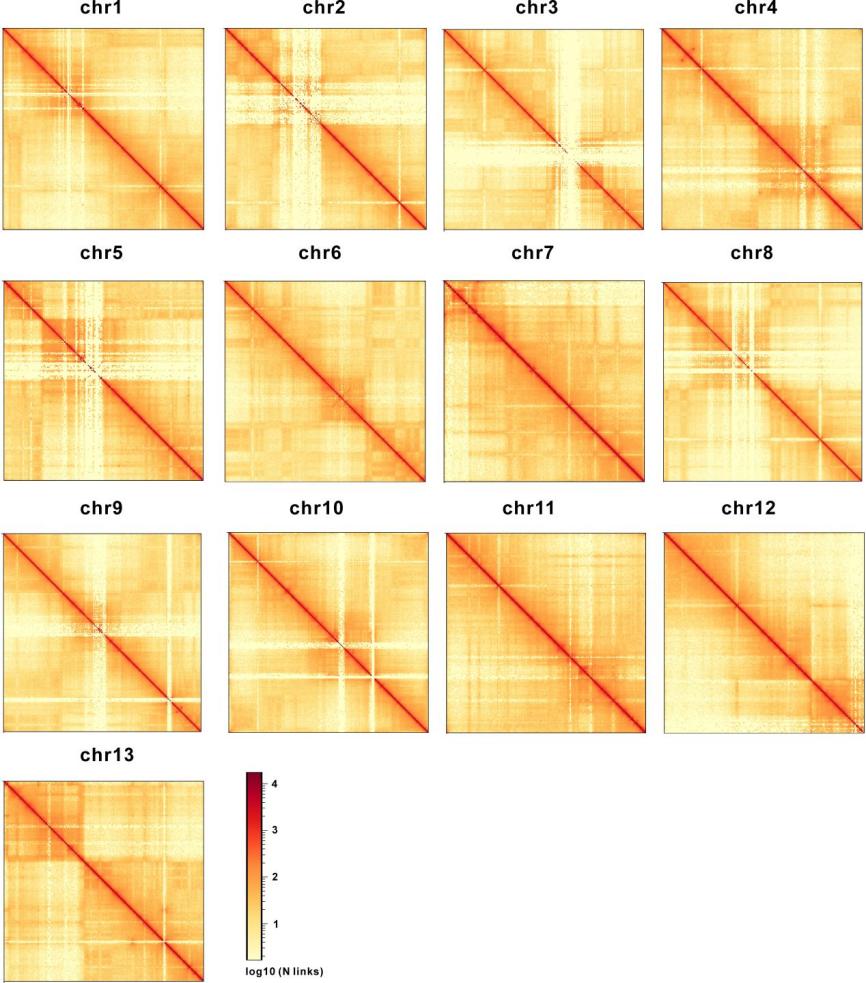


**Supplementary Figure S3. Heatmap showing Hi-C interactions of all sesame pseudochromosomes.**


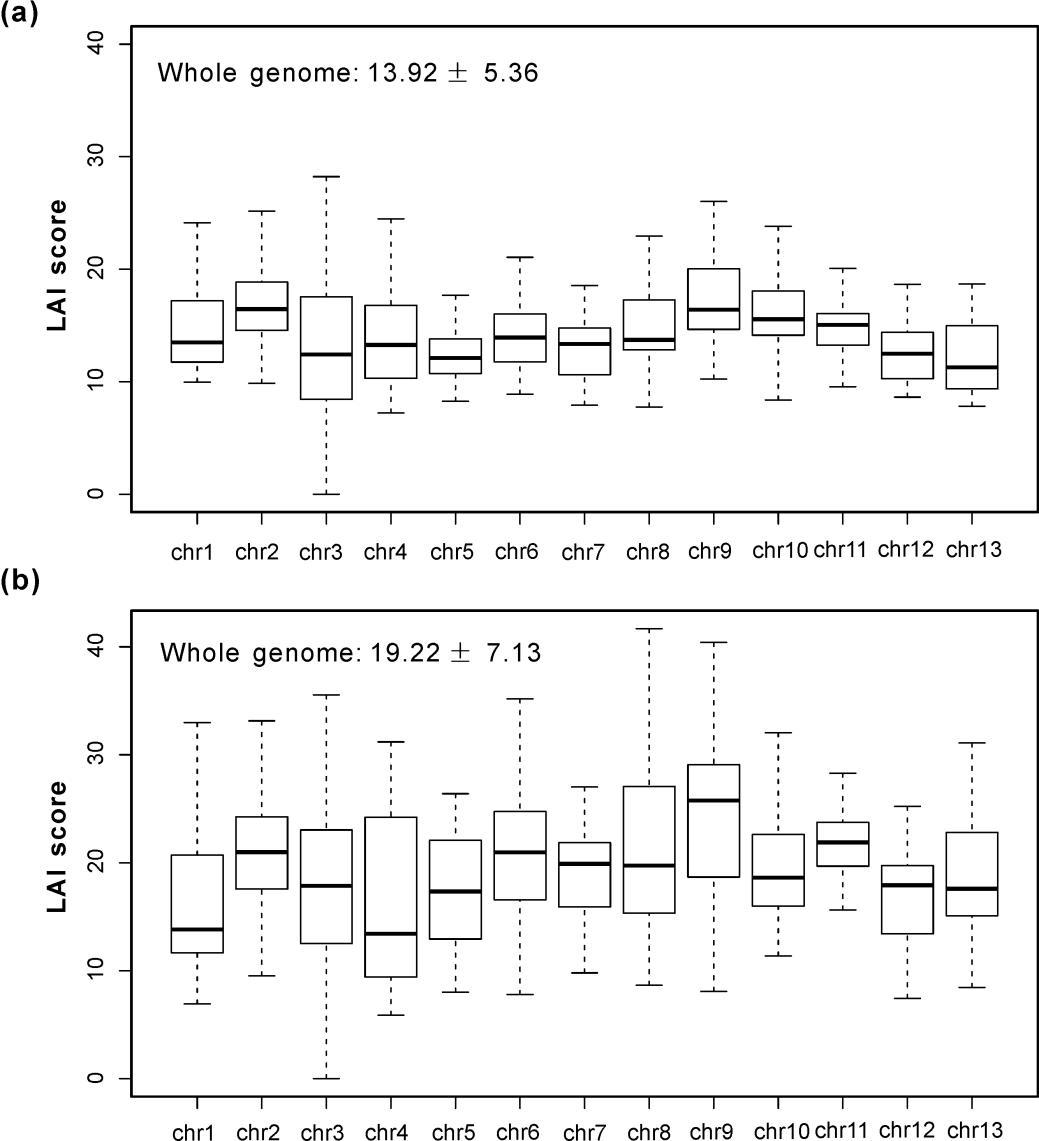


**Supplementary Figure S4. Distribution of LTR Assembly Index (LAI) score in all pseudochromosomes of previous (v2; a) and our updated (v3; b) sesame genome assemblies.**


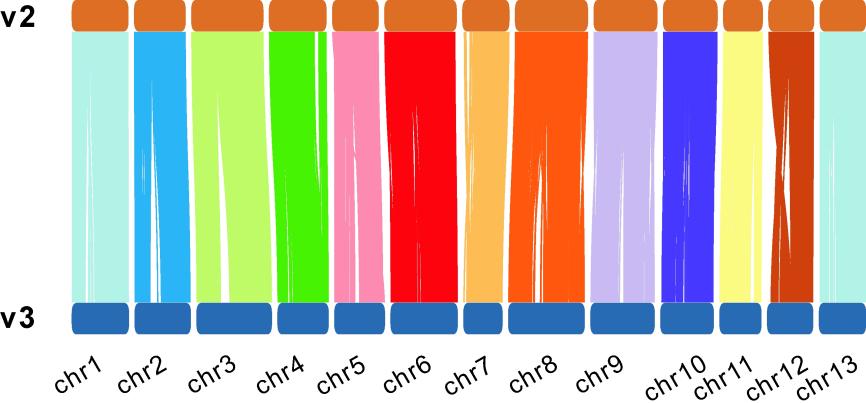


**Supplementary Figure S5. Global collinearity between the previous (v2) and our updated sesame genome (v3) assemblies obtained by MUMmer analysis.**


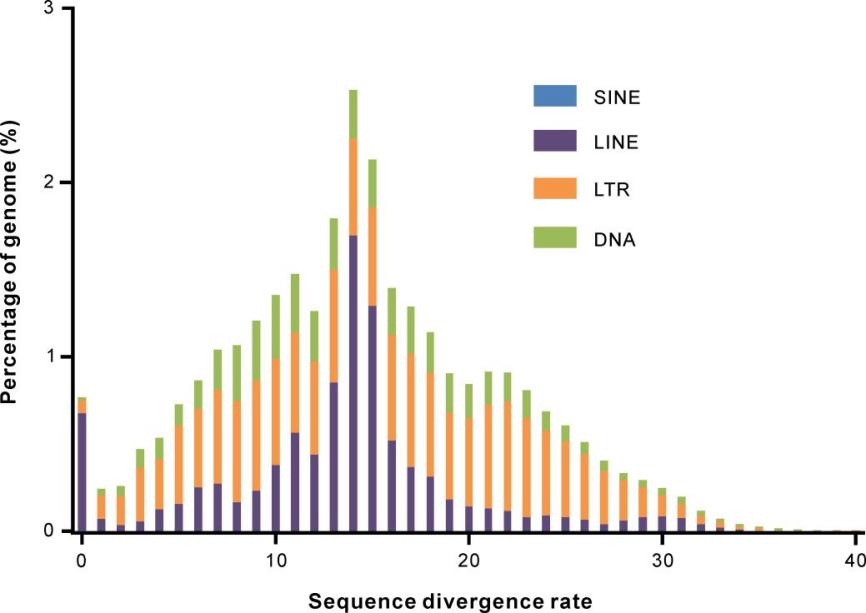


**Supplementary Figure S6. Distribution of divergence rate of different transposable element types in the sesame v3 assembly.**


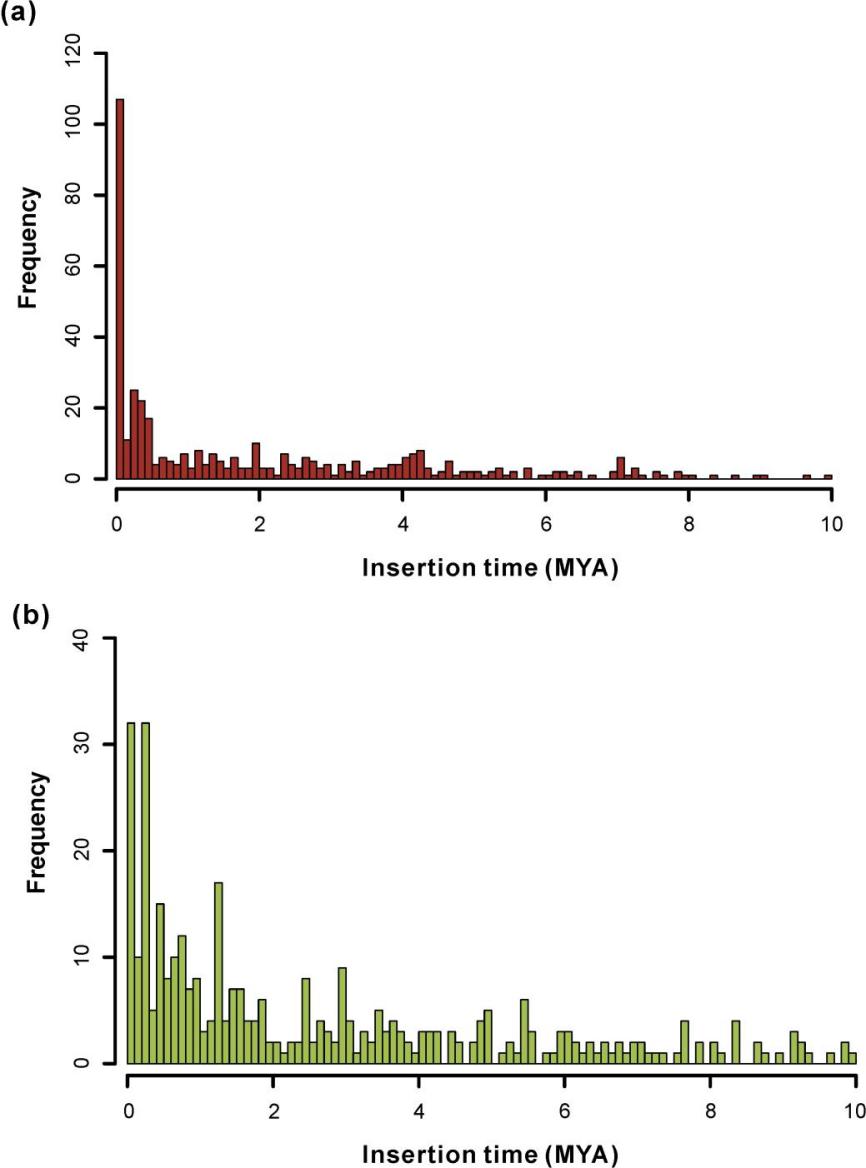


**Supplementary Figure S7. Insert age distribution of intact LTR-RTs (a: *Copia*; b: *Gypsy*) within the sesame v3 assembly.**


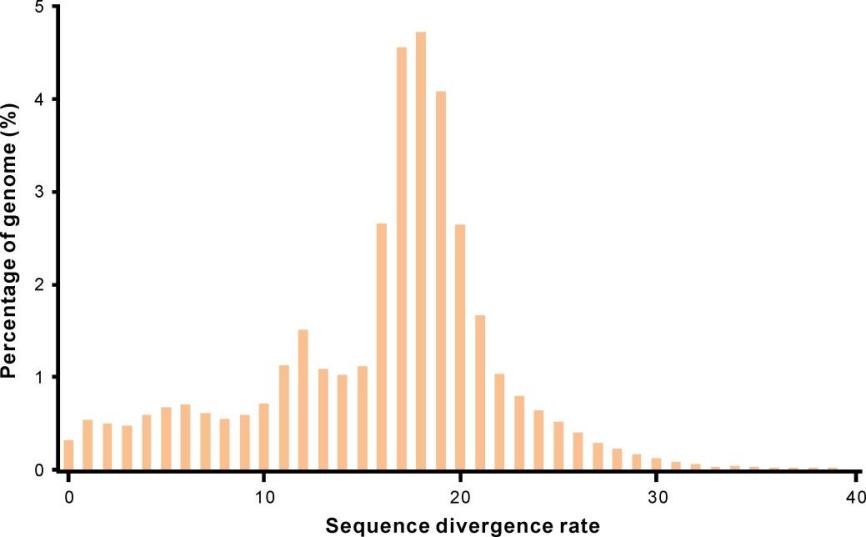


**Supplementary Figure S8. Distribution of divergence rate of unclassified repetitive sequences in the sesame v3 assembly.**


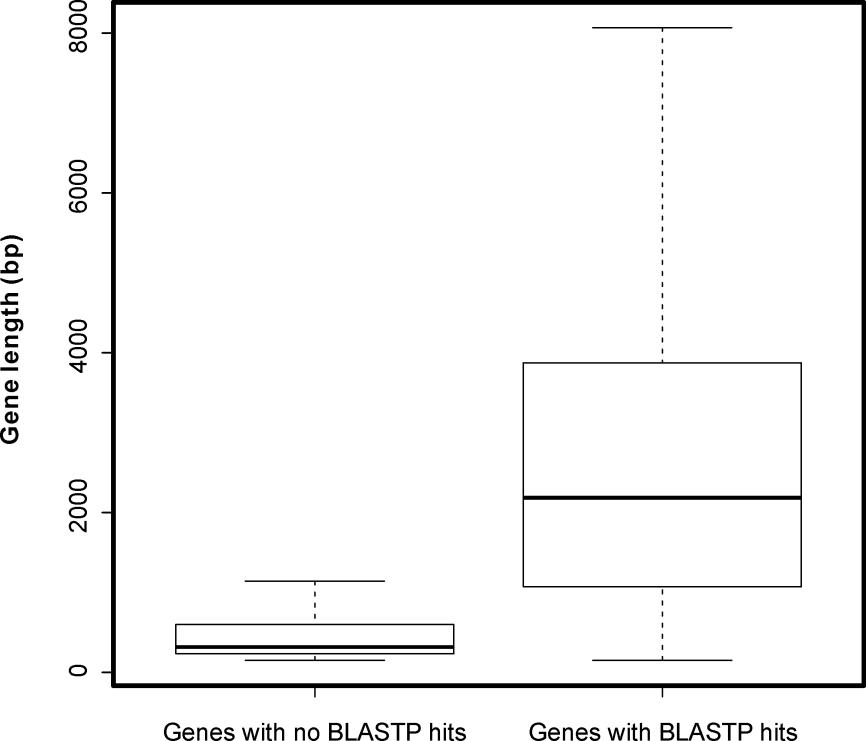


**Supplementary Figure S9. Gene length comparison between the genes with no BLASTP hits to our updated annotation set (v3) and other genes in the sesame v2 annotation set.**


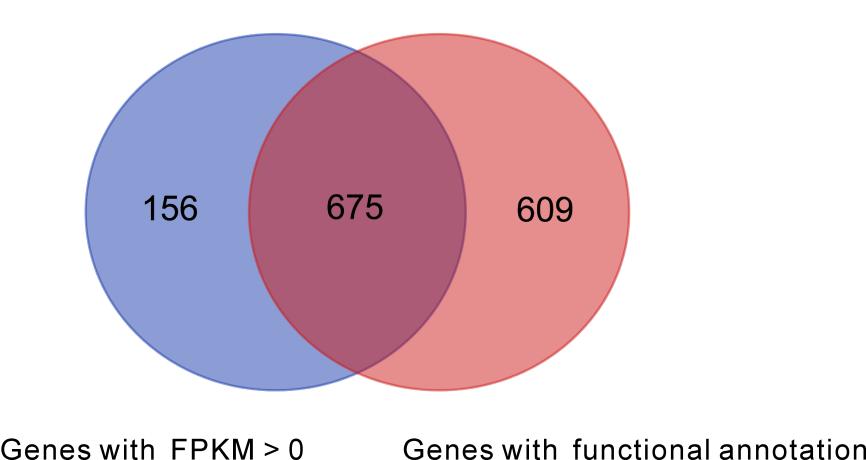


**Supplementary Figure S10. Venn diagram of the 1,440 genes supported by functional annotation or gene expression analysis.**


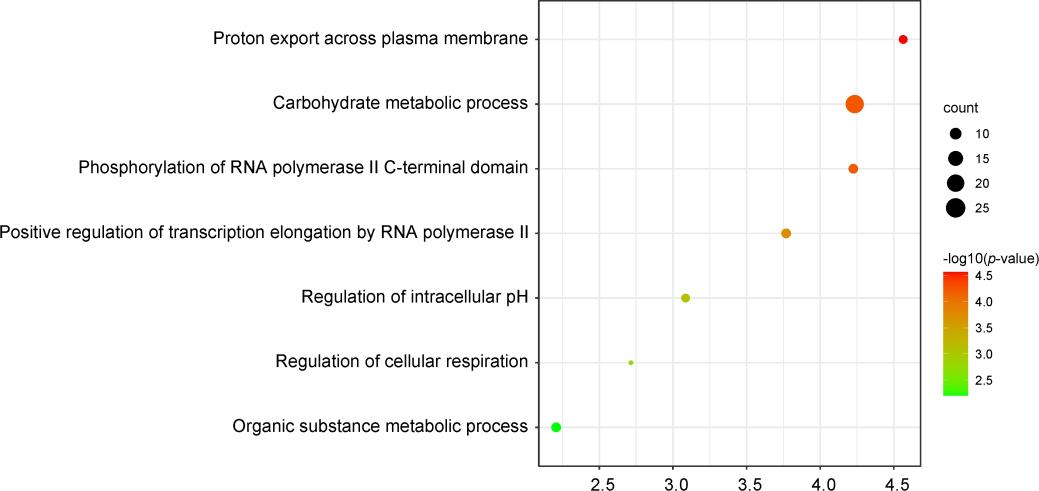


**Supplementary Figure S11. Enriched GO terms for the genes unique to sesame.**


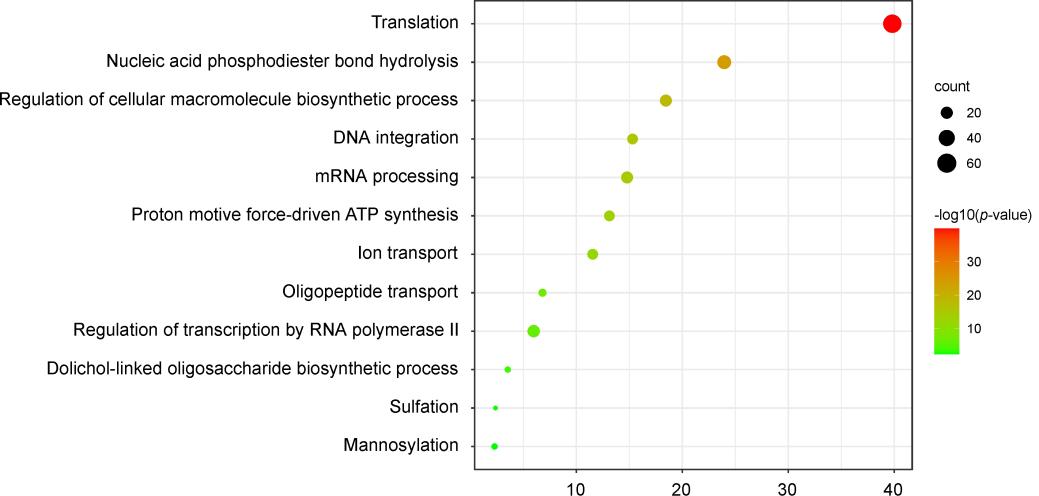


**Supplementary Figure S12. Enriched GO terms for the expanded gene families in sesame.**


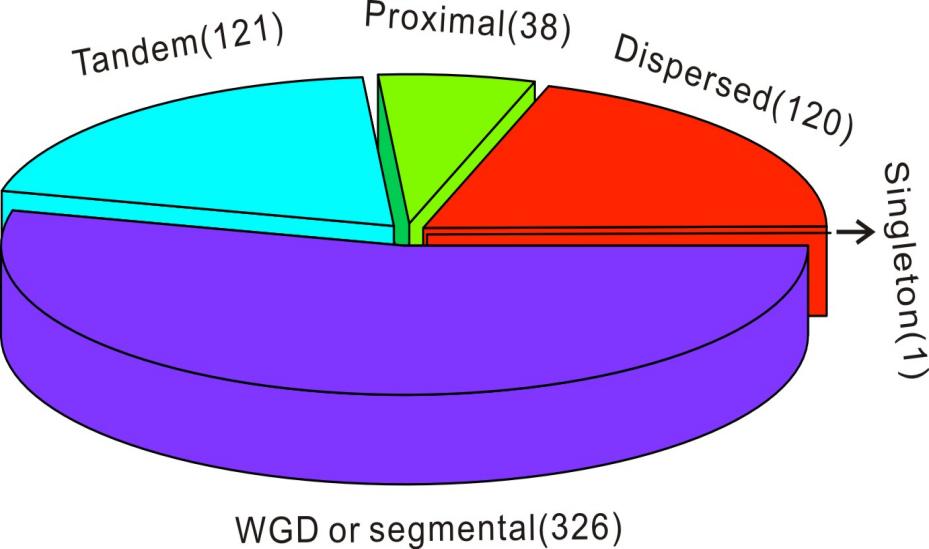


**Supplementary Figure S13. Different types of genes located in expanded gene families in sesame classified by MCScanX.**

**
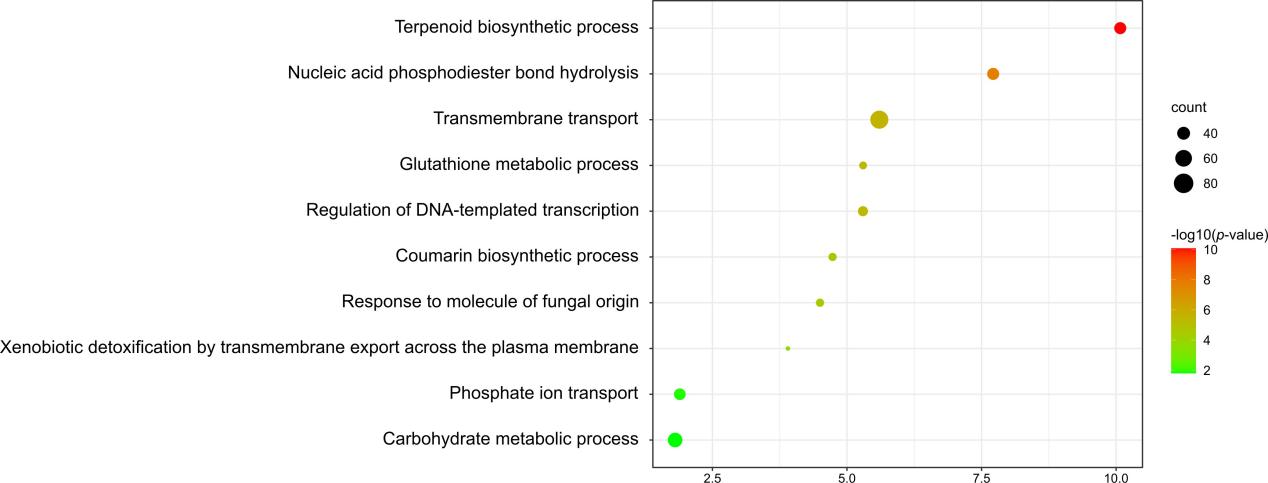
**

**Supplementary Figure S14. Enriched GO terms for the tandemly duplicated genes in sesame.**

| **Platform** | **Illumina** | **HiFi** | **Hi-C** |
| --- | --- | --- | --- |
| Read length (bp) | 150 | - | 150 |
| Number of reads | 81,094,260*2 | 2,011,950 | 107,785,669*2 |
| Number of bases (bp) | 24,328,278,000 | 34,329,379,444 | 32,335,700,700 |
| Sequence coverage (×)* | 76.74 | 108.29 | 102.00 |
| Accession number | SRX17389771 | SRX17602893 | SRX17602393 |

* The estimated genome size was ~317.02 Mb.

**Supplementary Table S1. Summary of the Illumina, PacBio’s HiFi and Hi-C data for the updated sesame assembly.**

|  | **v2** | | | | **v3** | | | |
| --- | --- | --- | --- | --- | --- | --- | --- | --- |
|  | **Contig** | | **Scaffold** | | **Contig** | | **Scaffold** | |
|  | **Size (bp)** | **Number** | **Size (bp)** | **Number** | **Size (bp)** | **Number** | **Size (bp)** | **Number** |
| N50 | 53,067 | 1,507 | 20,257,639 | 6 | 13,482,096 | 8 | 23,373,591 | 6 |
| N60 | 42,528 | 2,068 | 18,415,740 | 8 | 11,516,036 | 11 | 22,211,858 | 7 |
| N70 | 32,643 | 2,778 | 16,756,707 | 9 | 10,841,595 | 14 | 20,964,454 | 9 |
| N80 | 23,125 | 3,745 | 16,472,772 | 11 | 9,627,895 | 17 | 19,499,366 | 10 |
| N90 | 13,063 | 5,241 | 16,275,532 | 12 | 4,495,268 | 21 | 17,331,639 | 12 |
| Longest | 471,223 | - | 26,180,356 | - | 31,438,572 | - | 31,985,284 | - |
| Number > 1 Mb | - | 0 | - | 13 | - | 29 | - | 13 |
| Number > 5 Mb | - | 0 | - | 13 | - | 20 | - | 13 |
| **Total** | **266,369,918** | **14,525** | **272,734,981** | **4,449** | **309,341,402** | **170** | **309,354,609** | **135** |

**Supplementary Table S2. Summary of the previous (v2) and our updated (v3) sesame genome assemblies.**

| **Superscaffold** | **Length (bp)** | **# of contigs** | **# of genes** |
| --- | --- | --- | --- |
| chr1 | 23,747,441 | 1 | 1,974 |
| chr2 | 23,373,591 | 4 | 1,751 |
| chr3 | 31,438,572 | 1 | 2,352 |
| chr4 | 21,226,290 | 4 | 1,534 |
| chr5 | 20,964,454 | 7 | 1,241 |
| chr6 | 27,991,760 | 2 | 2,392 |
| chr7 | 16,119,130 | 3 | 1,357 |
| chr8 | 31,985,284 | 4 | 2,439 |
| chr9 | 26,739,103 | 4 | 2,691 |
| chr10 | 22,211,858 | 5 | 1,563 |
| chr11 | 17,331,639 | 2 | 1,402 |
| chr12 | 19,100,465 | 3 | 1,870 |
| chr13 | 19,499,366 | 4 | 1,381 |
| **Total** | **301,728,953** | **44** | **23,947** |

**Supplementary Table S3. Summary of pseudochromosomes of the sesame v3 assembly.**

|  | **Feature** | **Value** |
| --- | --- | --- |
| Reads | Mapping rate (%) | 99.57 |
|  | Average sequencing depth | 62.66 |
|  | Coverage (%) | 99.90 |
| Genome | Coverage at least 4× (%) | 99.59 |
|  | Coverage at least 10× (%) | 99.13 |
|  | Coverage at least 20× (%) | 98.15 |

**Supplementary Table S4. Assessment of genome coverage rate using Illumina short reads.**

| **Tissue** | **Number of reads** | **Total size (bp)** | **Mapping rate (%)** | **Accession number** |
| --- | --- | --- | --- | --- |
| Leaf | 218,290,664 | 8,070,494,672 | 95.00 | SRX17378544 |
| Stem | 232,755,816 | 8,600,247,292 | 93.30 | SRX17378547 |
| Flower | 274,066,856 | 10,124,747,609 | 94.24 | SRX17378546 |
| Seed | 193,148,144 | 7,141,724,593 | 96.44 | SRX17378548 |
| Husk | 265,841,048 | 9,836,772,966 | 94.30 | SRX17378545 |
| **Total** | 1,184,102,528 | 43,773,987,132 | 94.56 | **-** |

**Supplementary Table S5. Summary of RNA-seq data used in this study.**

| **Position** | **Type** | **Region (start - end)** | **Length (bp)** |
| --- | --- | --- | --- |
| chr1_head | CCCTAAA | 4 - 20,038 | 14,280 |
| chr1_tail | TTTAGGG | 23,729,582 - 23,747,409 | 13,797 |
| chr2_head | CCCTAAA | 4 - 52,489 | 14,014 |
| chr2_tail | TTTAGGG | 23,360,933 - 23,373,590 | 9,912 |
| chr3_head | CCCTAAA | 7 - 57,234 | 13,160 |
| chr3_tail | TTTAGGG | 31,422,596 - 31,438,547 | 11,207 |
| chr4_head | CCCTAAA | 5 - 35,668 | 14,966 |
| chr4_tail | TTTAGGG | 21,219,891 - 21,226,288 | 6,069 |
| chr5_head | CCCTAAA | 4 - 14,163 | 10,668 |
| chr5_tail | TTTAGGG | 20,944,801 - 20,964,449 | 16,548 |
| chr6_head | CCCTAAA | 32 - 31,699 | 9,163 |
| chr6_tail | TTTAGGG | 27,974,327 - 27,991,757 | 17,423 |
| chr7_head | TTTAGGG | 2,266,925 - 2,277,339 | 70 |
| chr7_tail | TTTAGGG | 16,084,513 - 16,119,127 | 16,247 |
| chr8_head | CCCTAAA | 92 - 21,140 | 9,891 |
| chr8_tail | TTTAGGG | 31,979,699 - 31,983,585 | 126 |
| chr9_head | CCCTAAA | 5 - 16,389 | 14,427 |
| chr9_tail | TTTAGGG | 26715170 - 26739097 | 11,802 |
| chr10_head | CCCTAAA | 2,161 - 18,384 | 294 |
| chr10_tail | TTTAGGG | 22,171,466 - 22,211,813 | 27,580 |
| chr11_head | - | Absent | - |
| chr11_tail | TTTAGGG | 17,309,812 - 17,331,638 | 18,242 |
| chr12_head | - | Absent | - |
| chr12_tail | TTTAGGG | 19,088,783 - 19,100,113 | 7,364 |
| chr13_head | TTTAGGG | 3,395,274 - 3,437,314 | 119 |
| chr13_tail | TTTAGGG | 19,477,274 - 19,499,361 | 20,440 |

**Supplementary Table S6. Distribution of telomere sequences in the sesame v3 assembly.**

| **Superscaffold** | **Start** | **End** | **Length (bp)** | **Repeat content (%)** |
| --- | --- | --- | --- | --- |
| chr1 | 5,439,383 | 6,878,048 | 1,438,666 | 90.65 |
| chr2 | 6,282,301 | 9,552,322 | 3,270,022 | 99.49 |
| chr2 | 9,582,271 | 11,131,708 | 1,549,438 | 99.83 |
| chr3 | 9,877,975 | 13,934,224 | 4,056,250 | 99.53 |
| chr5 | 8,485,137 | 10,501,574 | 2,016,438 | 97.11 |
| chr7 | 141,477 | 1,216,050 | 1,074,574 | 96.57 |
| chr8 | 10,921,085 | 13,435,525 | 2,514,441 | 99.53 |
| chr8 | 13,736,088 | 14,790,707 | 1,054,620 | 99.11 |
| chr9 | 12,049,373 | 13,944,068 | 1,894,696 | 99.92 |

**Supplementary Table S7. Location and repeat content of large blocks (longer than 1 Mb) in the v3 assembly that showed no similarity to any region of the v2 assembly.**

|  | **v2 assembly** | | **v3 assembly** | |
| --- | --- | --- | --- | --- |
| **Type** | **Total length (bp)** | **% of genome** | **Total length (bp)** | **% of genome** |
| DNA | 12,508,040 | 4.59 | 16,276,118 | 5.26 |
| LINE | 9,255,380 | 3.39 | 24,062,945 | 7.78 |
| SINE | 109,822 | 0.04 | 137,883 | 0.04 |
| LTR | 36,998,767 | 13.57 | 44,361,053 | 14.34 |
| *Gypsy* | 13,232,996 | 4.85 | 20,457,150 | 6.61 |
| *Copia* | 19,919,491 | 7.30 | 22,015,923 | 7.12 |
| Other | 3,846,280 | 1.41 | 1,887,980 | 0.61 |
| Satellite | 206,756 | 0.08 | 394,328 | 0.13 |
| Simple repeat | 53,281 | 0.02 | 57,786 | 0.02 |
| Low complexity | 16,952 | 0.01 | 4,637 | 0.00 |
| Unknown | 50,889,182 | 18.66 | 81,368,178 | 26.30 |
| **Total** | **109,801,638** | **40.26** | **163,380,884** | **52.81** |

**Supplementary Table S8. Classification of repetitive elements in the the previous (v2) and updated (v3) sesame genome assemblies.**

|  | **Number of genes** | **Percent (%)** |
| --- | --- | --- |
| Total | 24,345 | - |
| Annotated | 23,516 | 96.59 |
| InterPro | 23,051 | 94.68 |
| KEGG | 8,270 | 33.97 |
| Swiss-Prot | 15,522 | 63.76 |
| TrEMBL | 21,909 | 89.99 |
| GO | 18,722 | 76.90 |
| Unannotated | 829 | 3.41 |

**Supplementary Table S9.** **Functional annotation of the protein-coding genes in the sesame v3 assembly.**

| **TF family** | **Gene number** | **TF family** | **Gene number** | **TF family** | **Gene number** |
| --- | --- | --- | --- | --- | --- |
| AP2 | 13 | GRAS | 42 | NF-YC | 12 |
| ARF | 14 | GRF | 11 | NZZ/SPL | 3 |
| ARR-B | 8 | GeBP | 6 | Nin-like | 11 |
| B3 | 43 | HB-PHD | 2 | RAV | 3 |
| BBR-BPC | 6 | HB-other | 14 | S1Fa-like | 3 |
| BES1 | 8 | HD-ZIP | 32 | SAP | 3 |
| C2H2 | 79 | HRT-like | 2 | SBP | 21 |
| C3H | 41 | HSF | 3 | SRS | 10 |
| CAMTA | 2 | LBD | 47 | TALE | 7 |
| CO-like | 13 | LFY | 1 | TCP | 22 |
| CPP | 6 | LSD | 4 | Trihelix | 26 |
| DBB | 5 | M-type_MADS | 19 | VOZ | 2 |
| Dof | 9 | MIKC_MADS | 7 | WOX | 15 |
| E2F/DP | 9 | MYB | 125 | WRKY | 50 |
| EIL | 6 | MYB_related | 62 | Whirly | 2 |
| ERF | 69 | NAC | 43 | YABBY | 5 |
| FAR1 | 17 | NF-X1 | 2 | ZF-HD | 6 |
| G2-like | 38 | NF-YA | 10 | bHLH | 128 |
| GATA | 14 | NF-YB | 4 | bZIP | 58 |

**Supplementary Table S10. Classification of transcription factor genes in the sesame v3 assembly.**

| **Type** | **Number** | **Average length (bp)** | **Total length (bp)** |
| --- | --- | --- | --- |
| miRNA | 129 | 130.12 | 16,785 |
| tRNA | 1,488 | 75.08 | 111,724 |
| rRNA | 2,002 | 174.31 | 348,973 |
| 28S | 239 | 138.40 | 33,077 |
| 18S | 165 | 770.56 | 127,143 |
| 5.8S | 66 | 153.11 | 10,105 |
| 5S | 1,532 | 116.61 | 178,648 |
| snRNA | 350 | 121.59 | 42,557 |
| CD-box | 164 | 99.41 | 16,304 |
| HACA-box | 38 | 130.11 | 4,944 |
| Splicing | 148 | 143.98 | 21,309 |

**Supplementary Table S11. Summary of noncoding RNAs in the sesame v3 assembly.**

| **#Genes/array** | **#Array** | **#Gene** |
| --- | --- | --- |
| 2 | 629 | 1,258 |
| 3 | 148 | 444 |
| 4 | 47 | 188 |
| 5 | 17 | 85 |
| 6 | 12 | 72 |
| 7 | 10 | 70 |
| 8 | 1 | 8 |
| 9 | 2 | 18 |
| 10 | 2 | 20 |
| >10 | 18 | 619 |
| Total | 886 | 2,782 |
| % of all genes | 11.43 | |

**Supplementary Table S12. The tandemly duplicated gene arrays identified in the sesame v3 assembly.**
